# Music To My Eyes? Exploring the pupil old/new effect to familiar and unfamiliar music in people with logopenic variant Primary Progressive Aphasia

**DOI:** 10.1002/alz.084507

**Published:** 2025-01-09

**Authors:** Emilie V Brotherhood, Lucy Core, Benjamin Aaron Levett, Eleanor O'Neill, Nicholas C. Firth, Keir X X Yong, Jason D Warren, Sebastian J Crutch

**Affiliations:** ^1^ Dementia Research Centre, UCL Queen Square Institute of Neurology, University College London, London United Kingdom; ^2^ Department of Computer Science, University College London, London United Kingdom

## Abstract

**Background:**

Responses to individualized music in people living with dementia can be indicated by both verbal and non‐verbal cues. Evidence suggests that elevated pupil dilation responses to familiar vs. unfamiliar music are preserved in people living with typical Alzheimer’s disease (tAD), and to an extent in people with its atypical ‘visual’ variant (Posterior Cortical Atrophy; PCA) (Brotherhood et al., 2023). In the present work, we investigate whether this finding is also observable in individuals with the atypical ‘language‐led’ variant of Alzheimer’s disease, logopenic variant Primary Progressive Aphasia (lvPPA).

**Method:**

Individuals with lvPPA or tAD (and cognitively normal adults) are currently being recruited via the Specialist Cognitive Disorders clinic at the National Hospital for Neurology and Neurosurgery, London, UK. We are simultaneously recording in‐the‐moment multiple physiological responses (e.g. pupillometry, electrodermal activity) while each participant listens to 30 different musical clips (classical (5 pieces), 1960s (10 pieces), 1970s (10 pieces), and five reversed pieces). Each clip has an a priori familiarity rating (known/unknown), participant‐rated pleasantness and familiarity, and participant‐rated ability to evoke a memory (yes/no). We will collect complementary basic clinical, audiometry and music experience data. Alongside pupillometry, multistrand physiological data via Empatica E4 devices will enable exploration of potential differential cardiovascular (heart rate/heart rate variability) and/or electrodermal activity (EDA) responses to familiar and unfamiliar music using within‐group linear regression models with robust standard errors.

**Result:**

Physiological responses from a priori‐predicted and subjective ratings of music familiarity will be reported for each participant group, controlling for demographic factors including participant age and disease severity. Prospective results will provide further insight into what is currently known about pupillometry responses to familiar/unfamiliar music in atypical AD (PCA) and tAD (e.g. Estimated mean % increase in known vs unknown songs [95%CI]: Controls 1.8% [1.2, 2.5]; tAD 2.6% [0.6, 4.6]; PCA 2.0% [1.0, 4.0]).

**Conclusion:**

The results of this work will provide insight into the extent to which differential responses to music at the physiological level is retained within lvPPA. This may both support the notion of individualized music provision, and potentially provide a mechanism for these individuals to communicate their subjective experience of music using a non‐verbal mechanism.

